# Clinical Predictors for Early Aspiration in Patients With Liver Abscesses: Insights From a Combined-Risk Model

**DOI:** 10.7759/cureus.91047

**Published:** 2025-08-26

**Authors:** Jinal Thakkar, Gunvant Rathod, Vipul D Yagnik, Prema R Chaudhary, Foram Modh

**Affiliations:** 1 General Surgery, Banas Medical College and Research Institute, Palanpur, IND; 2 General Surgery, Dr. M. K. Shah Medical College and Research Centre, Ahmedabad, IND; 3 Surgery, Banas Medical College and Research Institute, Palanpur, IND; 4 Physiology, Banas Medical College and Research Institute, Palanpur, IND

**Keywords:** amoebic liver abscess, combined-risk model, liver abscess, percutaneous aspiration, predictive factors

## Abstract

Background: Liver abscesses represent a significant contributor to surgical morbidity, particularly in developing nations, with amoebic liver abscess (ALA) being the most common type. The debate surrounding the role of early aspiration in preventing complications makes this study particularly important.

Methods: This prospective observational study included 30 consecutive adult patients diagnosed with liver abscesses through radiological imaging over a two-year period at a tertiary care hospital in India. Patients who had ruptured abscesses necessitating surgical intervention were excluded to avoid confounding effects on predictive factors and the timing of aspiration. Clinical and imaging data, including age, sex, duration of symptoms, number and size of abscesses, and lobe involvement, were collected. Aspiration was categorized as early (≤4 days) or late (>4 days). Statistical associations were evaluated using chi-square/Fisher’s exact test, and odds ratios (OR) were computed.

Results: The study primarily comprised male patients (n=24, 80.0%), with a mean age of 47.6 years. ALA constituted n=29, 96.57% of the cases. No individual factor showed a statistically significant correlation with early aspiration (p>0.05). However, among patients with two or more moderate-risk factors, defined here as age ≥55 years, abscess size ≥5 cm, and bilateral involvement, there was a trend toward the need for early aspiration (n=6/10, 60.0% vs. n=5/20, 25.0%, OR=4.5, p=0.08).

Conclusion: In studies involving small, homogeneous samples, the statistical power of individual predictors may be limited. A combined-risk model could provide a more accurate method of identifying candidates for early aspiration. Nonetheless, the urgency of this issue is clear, indicating the necessity for validation through larger, multi-center studies to enhance treatment strategies for liver abscesses.

## Introduction

Liver abscesses account for approximately 48% of all visceral abscesses globally, making them the most prevalent type [1]. The reported prevalence varies from 44 to 80% [2,3]. This condition is particularly common in low- and middle-income countries, where infections caused by Entamoeba histolytica are endemic [4,5]. In these areas, amoebic liver abscess (ALA) is the predominant type, whereas pyogenic liver abscess (PLA) is more frequently observed in developed countries, often associated with biliary disease or systemic sepsis [6].

Standard management typically involves a combination of antimicrobial therapy and image-guided aspiration or catheter drainage in specific cases, serving diagnostic, therapeutic, or prophylactic purposes [7,8]. A large-scale study with 966 patients identified independent predictors for early aspiration, including age ≥55 years, abscess size ≥5 cm, symptom duration ≥7 days, and bilateral lobe involvement [9]. However, these factors are often analyzed in isolation, and there is limited understanding of the predictive significance of multiple moderate-risk factors when considered together. The primary objective is to evaluate the association between predefined clinical/imaging predictors (age, symptom duration, abscess size, multiplicity, lobe involvement) and the need for early aspiration (≤4 days) and the secondary objective is to assess a prespecified combined-risk rule (≥2 of age ≥55, abscess diameter ≥5 cm, bilateral involvement) and estimate its effect size (OR) and precision. This study aims to explore traditional predictors within a homogeneous cohort predominantly affected by ALA in India. It also seeks to determine whether a composite risk assessment can enhance the prediction of the need for early aspiration.

## Materials and methods

Study design and setting

This was a prospective observational cohort study conducted over 24 months, from January 2023 to December 2024, at SMS Multispeciality Hospital and M. K. Shah Medical College and Research Centre in Ahmedabad, Gujarat, India, a tertiary care referral center. The study aimed to identify clinical and imaging predictors of early aspiration in patients with liver abscesses, focusing on creating a combined moderate-risk factor model tailored to an ALA-predominant population.

Study objectives and hypotheses

The primary objective of this study was to evaluate the association between predefined clinical and imaging predictors (age, symptom duration, abscess size, multiplicity, and lobe involvement) and the need for early aspiration, defined as image-guided percutaneous aspiration within four days of admission.

The secondary objective was to assess a prespecified combined-risk rule, where early aspiration was considered more likely when ≥2 of the following factors were present: age ≥55 years, abscess diameter ≥5 cm, and bilateral involvement.

We hypothesized that (i) individual clinical and imaging predictors would be associated with early aspiration (≤4 days), and (ii) the combined-risk rule would demonstrate a stronger association with early aspiration than any single predictor alone. Outcomes and thresholds were defined a priori based on institutional practice and prior literature.

Patient recruitment

**Figure 1:**
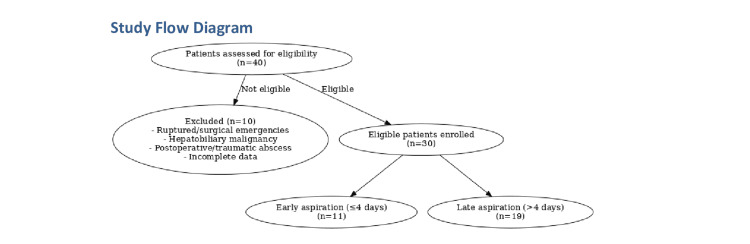
Flow diagram showing patient screening (n=40), exclusions (n=10), and allocation to early (≤4 days, n=11) and late (>4 days, n=19) aspiration groups

All consecutive patients admitted with a primary diagnosis of liver abscess during the study period were screened for eligibility by the attending surgical and radiology teams within the first 24 hours of admission. No randomization or intervention allocation was performed; management decisions were entirely at the discretion of the treating team. Patients who met the inclusion criteria were enrolled after providing informed written consent. A total of 40 patients were screened for eligibility, of whom 10 were excluded (ruptured/surgical emergencies, hepatobiliary malignancy, postoperative/traumatic abscess, incomplete data). Thirty patients were enrolled and analyzed, with 11 undergoing early aspiration (≤4 days) and 19 late aspiration (>4 days). The study flow is summarized in Figure 1.

Inclusion and exclusion criteria

The inclusion criteria comprised patients aged more than 18 years at the time of admission, those with a radiologically confirmed liver abscess diagnosed using abdominal ultrasonography or contrast-enhanced computed tomography, and those whose initial management plan included conservative therapy with broad-spectrum intravenous antibiotics and supportive treatment. The exclusion criteria included patients with clinical or imaging signs of a ruptured liver abscess requiring immediate surgical intervention, those with known hepatobiliary malignancies such as cholangiocarcinoma or hepatocellular carcinoma, those with secondary liver abscesses resulting from trauma or postoperative complications, and those lacking demographic, clinical, or imaging data.

Diagnostic strategy

Upon admission, all patients underwent a baseline abdominal ultrasound. In cases where the ultrasound was non-diagnostic or there were suspicions of bilobar or multiple abscesses or rupture, a contrast-enhanced computed tomography scan was performed. Imaging parameters evaluated included the number of abscess cavities (solitary, multiple, or unknown), the maximum diameter of the largest abscess measured in centimeters in the axial plane on ultrasonography or computed tomography, the lobe involvement (right, left, or bilateral), and any associated features such as imminent rupture, peritoneal free fluid, or subdiaphragmatic extension. Maximum diameter was used as a surrogate for volume, as it is directly proportional to abscess size in near-spherical lesions and is more practical for routine clinical use. Volumetric assessment requires advanced software and is often not feasible in resource-limited settings. Moreover, prior studies have shown that maximum diameter strongly correlates with abscess volume, making it a reliable indicator for clinical decision-making.

The classification of abscesses into ALA and PLA was based on the clinical presentation, including history of dysentery and endemic exposures, the imaging characteristics, serological testing for E. histolytica IgG antibodies when indicated, and the exclusion of bacterial origin through negative blood cultures where obtainable. IgG testing for Entamoeba histolytica was obtained when clinical suspicion of amoebic etiology was high, blood cultures were negative or non-diagnostic, or imaging features were inconclusive. Assays followed manufacturer‑specified cut‑offs; positive serology in the appropriate clinical context supported the ALA diagnosis.

Aspiration protocol

All aspirations were performed under real-time ultrasound guidance using a 16-G BD spinal needle. A single-pass technique was employed in each case, with repeat aspiration only if clinical improvement was not observed within 72 hours. All patients received intravenous broad-spectrum antibiotics before and for at least 48 hours after the procedure, along with appropriate analgesia and monitoring for complications.

Clinical criteria for early aspiration

In addition to the predefined threshold of ≤4 days from admission, early aspiration was considered in patients exhibiting persistent fever (>38°C), worsening right upper quadrant pain, or lack of clinical response to antibiotic therapy within 72 hours of initiation. This ensured timely intervention in clinically deteriorating patients while maintaining consistency with institutional practice.

Data collection

A standardized data collection sheet was used to record demographic characteristics (age, sex, occupation, and residential location as urban or rural), clinical presentation (onset and duration of symptoms, leading symptoms such as right upper quadrant pain, fever, jaundice, anorexia, and weight loss, and comorbidities including diabetes mellitus, chronic liver disease, immunosuppression, and alcohol use), laboratory parameters (complete blood count, liver function tests, renal function tests, C-reactive protein, erythrocyte sedimentation rate, and blood cultures), and treatment information (timing of aspiration defined as early or late, type of procedure, antibiotic treatments administered, and any escalation of therapy required).

Definition of early aspiration

Early aspiration was defined as image-guided percutaneous aspiration performed within four days of admission, whereas late aspiration referred to those carried out after the four-day mark. We selected a ≤4-day threshold based on local workflow and resource availability. Although previous studies have used a day-5 threshold for early aspiration [9], our approach minimizes ambiguity and aligns with institutional practices. We performed a post-hoc sensitivity analysis comparing ≤4 days vs ≤5 days; effect estimates were directionally unchanged, and differences were not statistically significant.

Statistical analysis

Data were entered into Microsoft Excel and analyzed using IBM SPSS Statistics for Windows, Version 25 (Released 2017; IBM Corp., Armonk, New York, United States). Categorical variables were expressed as frequencies and percentages, while continuous variables were reported as mean ± standard deviation for normally distributed data or as median with interquartile range for skewed data. Comparisons of categorical variables were performed using the Chi-square test or Fisher’s exact test, as appropriate. Continuous variables were assessed for normality using the Shapiro-Wilk test. Comparisons between Early (≤4 days) and Late (>4 days) aspiration groups were performed using Student’s t-test or Mann-Whitney U test for continuous variables and Chi-square or Fisher’s exact test for categorical variables. Odds ratios with 95% confidence intervals were calculated to determine the strength of association of predictors with early aspiration. A p-value of less than 0.05 was considered statistically significant, and all tests were two-tailed. The study was not powered for multivariable modeling due to the limited number of events; therefore, analyses were exploratory and hypothesis-generating.

Ethical considerations

The Institutional Ethics Committee of SMS Multispeciality Hospital and Dr M.K. Shah Medical College and Research Centre, with approval number MKSMCRC/IEC/SERC/2146, approved the protocol. Written consent was obtained from all participants before enrollment. Patient confidentiality was strictly maintained, and all data were anonymized before analysis.

## Results

Patient demographics and clinical features

Thirty patients were included according to the inclusion criteria. The male gender predominated (n=24, 80.0%), with a mean age of 47.63 ± 11.8 years (range 22-68 years); nine patients (30.0%) were above 55 years of age. ALA was the predominant etiology, seen in 29 patients (96.57%), whereas PLA was observed in only one patient (3.33%). Right upper quadrant pain was the most frequent presenting symptom (n=27, 89.9%), followed by fever (n=21, 69.9%), anorexia (n=13, 43.3%), nausea/vomiting (n=10, 33.3%), and jaundice (n=3, 10.0%). Seventeen patients (56.6%) experienced symptom duration for more than seven days. Comorbidities occurred in a proportion of patients, with diabetes mellitus being the most prevalent (n=7, 23.3%), followed by chronic liver disease (n=2, 6.6%) and a history of alcohol use (n=8, 26.6%).

On laboratory assessment, leukocytosis (>11,000/cmm) was present in 19 patients (63.3%), elevated CRP in 18 of 28 tested patients (60.0%), and abnormal liver function tests in 14 patients (46.6%). Imaging revealed single abscesses in 22 patients (73.3%), most commonly located in the right lobe (n=23, 76.6%). Multiple abscesses were seen in eight patients (26.7%), and bilateral disease in three patients (10.0%). The maximal abscess diameter was 6.1 ± 1.4 cm, with 21 patients (70.0%) having a diameter of ≥5 cm (Table 1).

**Table 1:** Baseline demographic, clinical, laboratory, and imaging characteristics of study participants (n = 30) Data are presented as number (percentage) unless otherwise specified. Percentages are calculated from total study population (n=30) unless indicated. Laboratory cut-offs based on institutional norms.

Characteristic	n (%) / Mean ± SD	Notes
Age (years)	47.63 ± 11.8	Range 22–68
Age ≥55 years	9 (30.0%)	
Male sex	24 (80.0%)	
Female sex	6 (20.0%)	
Amoebic liver abscess (ALA)	29 (96.57%)	
Pyogenic liver abscess (PLA)	1 (3.33%)	
Right upper quadrant pain	27 (89.9%)	
Fever	21 (69.9%)	
Anorexia	13 (43.3%)	
Nausea/vomiting	10 (33.3%)	
Jaundice	3 (10.0%)	
Symptom duration >7 days	17 (56.6%)	
Diabetes mellitus	7 (23.3%)	
Chronic liver disease	2 (6.6%)	
Alcohol use history	8 (26.6%)	
Leukocytosis (>11,000/cmm)	19 (63.3%)	
Elevated CRP (>10 mg/L)*	18 (60.0%)	Tested in 28 patients
Abnormal liver function tests	14 (46.6%)	
Single abscess	22 (73.3%)	
Multiple abscesses	8 (26.7%)	
Right lobe involvement	23 (76.6%)	
Left lobe involvement	4 (13.3%)	
Bilateral involvement	3 (10.0%)	
Abscess diameter ≥5 cm	21 (70.0%)	
Maximal abscess diameter (cm)	6.1 ± 1.4	

Predictive early markers of aspiration

When evaluating individual clinical and imaging parameters for their association with early aspiration (≤4 days after admission), no single factor achieved statistical significance. The presence of multiple abscesses (n=4, 50.0%, OR 2.00, p=0.657) and symptom duration greater than seven days (n=8, 47.0%, OR 1.53, p=0.638) indicated a non-significant trend towards early aspiration (Table 2).

**Table 2:** Predictive factors for early aspiration in liver abscess (n = 30) Percentages are calculated from the total study population (n=30). p-values obtained using Chi-square test or Fisher’s exact test, as appropriate.

Clinical factor	Overall cases n (%)	Early aspiration cases n (%)	Odds ratio (95% CI)	p-value
Age ≥55 years	9 (30.0%)	3 (33.3%)	1.20 (0.23–6.24)	0.708
Symptom duration >7 days	17 (56.6%)	8 (47.0%)	1.53 (0.37–6.31)	0.638
Abscess diameter ≥5 cm	21 (70.0%)	9 (42.8%)	1.09 (0.23–5.07)	0.935
Presence of multiple abscesses	8 (26.6%)	4 (50.0%)	2.00 (0.39–10.1)	0.657

Combined moderate-risk factor model

When patients were categorized based on having ≥2 moderate-risk factors (age ≥55 years, abscess diameter ≥5 cm, and bilateral hepatic involvement), early aspiration occurred more frequently in this group (n=6/10, 60.0%) compared to those with fewer than two factors (n=5/20, 25.0%). This yielded an odds ratio of 4.50 (p=0.080). Although not statistically significant, the findings suggest the potential clinical value of a combined-risk strategy (Table 3).

**Table 3:** Association between combined moderate-risk factors (>2 factors) and early aspiration Moderate-risk factors include age ≥55 years, abscess diameter ≥5 cm, and bilateral hepatic involvement. Percentages are calculated from the number of patients in each category. p-values obtained using Fisher’s exact test.

Presence of ≥2 moderate-risk factors	n	Early aspiration n (%)	OR (95% CI)	p-value
Present	10	6 (60.0%)	4.50 (0.78–25.9)	0.08
Absent	20	5 (25.0%)	–	–

## Discussion

In this prospective observational study focused on predominantly ALA, we found that no individual clinical or imaging predictors, such as age ≥55 years (n=9, 30.0%), symptoms lasting more than seven days (n=17, 56.6%), abscess diameter ≥5 cm (n=21, 70.0%), multiplicity (n=8, 26.6%), or bilobar involvement (n=3, 10.0%), were significantly associated with the need for early aspiration, defined as within ≤4 days of presentation. However, a multimodal moderate-risk factor model, utilizing at least two of the following, age ≥55 years, abscess size ≥5 cm, and bilateral hepatic involvement, indicated more than a two-fold increase in early aspiration occurrences (n=6/10, 60.0% vs. n=5/20, 25.0%), with an odds ratio of 4.50, although this did not reach statistical significance (p=0.080).

This outcome suggests that in a relatively uniform patient population, the predictive capability of individual variables may be limited, whereas the accumulation of risks can enhance discriminatory ability. The concept of risk factor aggregation has previously proven valuable in various infectious diseases, including invasive candidiasis [10] and severe community-acquired pneumonia, where multiple moderate predictors yield a higher positive predictive value than any single factor. This supports the potential utility of our combined moderate-risk model in clinical settings.

The epidemiological characteristics of our cohort, predominantly male (n=24, 80.0%) [11,12], with a mean age in the fifth decade [13,14], and a high occurrence of ALA (n=29, 96.57%), correspond with findings from significant studies in India and other tropical regions. This alignment strengthens our study's validity. Notably, previous research indicates that ALA accounts for 80-90% of hepatic abscess cases in endemic areas, with increased involvement of the right lobe due to various hydrodynamic factors [6]. The male predominance can be attributed to factors such as differential iron metabolism and increased exposure to risk behaviors like alcohol use and inadequate sanitation [15,16], and more recent molecular research supports the idea that the virulence of E. histolytica relies on iron, potentially explaining its higher incidence and severity in endemic regions, particularly among men [17].

Our findings contrast with those of Khan et al. [9], who identified abscess size ≥5 cm and bilobar involvement as independent predictors of early aspiration in a multicenter study of 966 patients. The differences may be attributed to several factors, including sample size, as our cohort of n=30 was insufficiently powered to establish individual predictor significance; population composition, since our study primarily focused on ALA, whereas Khan’s study included more PLAs, which are often more systemically inflamed and may require earlier intervention; and management protocols, as variability in institutional aspiration thresholds can influence statistical outcomes.

Recent studies on PLA have identified additional predictors for intervention timing. A Chinese multicenter survey from 2025 noted that specific clinical parameters, such as pneumonia, diabetes, and left-lobe disease, could predict abscess liquefaction amenable to drainage [18]. Another 2025 article in Frontiers in Medicine reported a sepsis risk nomogram integrating various laboratory parameters that demonstrated strong diagnostic discrimination [19]. These advancements highlight the growing trend towards multivariate predictive modeling for liver abscess management, reinforcing our findings’ potential in enhancing clinical decision-making, particularly in resource-limited settings. This composite approach parallels risk stratification models used in other infectious diseases, such as invasive candidiasis [10].

From a mechanistic perspective, advanced age [20] may hinder host defense effectiveness, larger abscesses typically harbor a greater bacterial or parasite load and exhibit increased intracavitary pressure, and bilobar involvement might indicate hematogenous spread or aggressive disease characteristics. The presence of two or more of these factors likely signifies more advanced or severe disease, explaining the increased urgency to prevent complications like rupture or systemic sepsis.

Strengths and limitations

The prospective design ensured even data capture and reduced recall bias, allowing for maximal reliability in clinical and imaging data. Endemic-specific analysis became feasible due to the homogeneous study group, composed predominantly of cases with ALA. Importantly, presenting a novel combined-risk model, based on the criterion of two or more moderate-risk factors as an alternative tool for predicting early aspiration need, introduces a new predictive methodology. Statistical analysis with odds ratios, confidence intervals, and p-values provides inferential width uncommon in small observational research, and the findings have clear clinical relevance for informing prioritization of aspiration in resource-limited health settings. However, due to the relatively modest sample size (n=30), statistical power to detect significant associations at the level of single predictors is limited. Due to the small sample size (n=30) and limited number of events, conducting extensive bivariate and multivariable analyses was not statistically appropriate as it would risk overfitting and generating unstable estimates. Therefore, we restricted our analysis to clinically relevant variables and presented results as exploratory, hypothesis-generating findings rather than definitive predictive modeling. Since it is a single-center study done in an ALA-predominant population, results may not generalize easily to areas where pyogenic liver abscess is predominant. Furthermore, because inflammatory biomarkers such as procalcitonin were not included in the evaluation, predictive analysis may have been limited in scope. The early aspiration definition of ≤4 days was based on our institutional protocol rather than a universally accepted standard, which may limit generalizability to other settings; however, a sensitivity analysis against a day-5 cut-off showed no material change in results, and long-term follow-up did not occur to gauge recurrence, resolution of cavity at sites persisting after presumed cure, or late complications.

Implications for clinical practice

In resource-constrained environments, indiscriminate early aspiration for all liver abscess cases is not feasible due to procedural risks such as bleeding, secondary infection, and bile leakage, as well as limited interventional radiology capacity. Although not statistically significant, the observed higher frequency of early aspiration among patients with ≥2 moderate-risk factors (60.0% vs. 25.0%; OR 4.50, p=0.080) suggests a potential role for a combined-risk model as a hypothesis-generating triage tool, warranting validation in larger cohorts. Moreover, embedding this model within electronic medical record decision-support systems could standardize practice, and reduce clinician variability. Similar initiatives in the management of sepsis and pneumonia have yielded improved outcomes and more efficient resource utilization.

## Conclusions

In this prospective observational cohort of patients with ALA, no individual clinical or imaging factor emerged as an independent predictor for early aspiration. A cumulative-risk approach incorporating multiple moderate-risk features appeared more informative than relying on single variables, suggesting potential value for risk stratification. These observations, while promising, are exploratory and should be validated through larger, multicenter studies with standardized protocols and external confirmation before being adopted in clinical practice.
